# HMGB1 Promotes Lymphangiogenesis through the Activation of RAGE on M2 Macrophages in Laryngeal Squamous Cell Carcinoma

**DOI:** 10.1155/2022/4487435

**Published:** 2022-03-04

**Authors:** Caili Su, Shuangshuang Jia, Zhihong Ma, Hong Zhang, Li Wei, Honggang Liu

**Affiliations:** ^1^Department of Pathology, Beijing Friendship Hospital, Capital Medical University, Beijing 100730, China; ^2^Department of Pathology, Beijing Tongren Hospital, Capital Medical University, Beijing Key Laboratory of Head and Neck Molecular Diagnostic Pathology, Beijing 100730, China

## Abstract

**Background:**

Receptor for advanced glycation end products (RAGE) is implicated in tumor biology. Released high mobility group box protein 1 (HMGB1) ligand binding to RAGE receptor in tumor cells promotes tumor progression. The mechanisms of HMGB1-RAGE signaling in M2 macrophages involved in lymphangiogenesis in laryngeal carcinoma remain poorly understood. Here, we assessed the effect of HMGB1-RAGE signaling on M2 macrophages in lymphangiogenesis.

**Methods:**

HMGB1, CD163, and D2-40 in laryngeal squamous cell carcinoma (LSCC, *n* = 123), laryngeal precursor lesions (LPLs, *n* = 102), and vocal polyp (VP, *n* = 55) were analyzed by immunohistochemistry. THP-1 cell-expressed RAGE gene was knocked down and then polarized to M0 macrophages and M2 macrophages. IL-23, TNF-*α*, TGF-*β*, and IL-10 were measured by ELISA; IL-1*β*, IL-12, IL-10, and CCL-13 were evaluated by RT-qPCR, and CD206, CD163, and RAGE were evaluated by western blot to evaluate whether classical M2 macrophages were obtained. Conditioned media from RAGE^+/-^ M0 macrophages and RAGE^+/-^ M2 macrophages incubated in the presence or absence of HMGB1, anti-Toll-like receptor (TLR)2, anti-TLR4 antibodies, and anti-VEGF-C antibodies were collected separately for human dermal lymphatic endothelial cells (HDLEC) for proliferation, migration, lymphangiogenesis assay, and VEGF-C concentration analysis.

**Results:**

HMGB1 and M2 macrophage densities were increased in LSCC (*P* < 0.01). HMGB1 and M2 macrophage densities were significantly correlated with lymphatic vessel density (LVD) in LSCC (*P* < 0.01). The HMGB1 overexpression and higher M2 macrophage density were involved in lymph node metastasis (*P* < 0.01) and poor prognosis (*P* < 0.05). *In vitro*, conditioned medium from HMGB1-stimulated RAGE^+^ M2 macrophages activated lymphangiogenesis by upregulating the VEGF compared to controls (*P* < 0.05). On the contrary, RAGE knockdown obviously decreased the corresponding effects of HMGB1-preconditioned M2 macrophages upon HDLEC (*P* < 0.05). HMGB1-TLR pathway does not significantly increase HDLEC proliferation, migration, and lymphangiogenesis on M2 macrophages.

**Conclusions:**

HMGB1 promotes lymphangiogenesis by activation of RAGE on M2 macrophages. Targeting RAGE may provide an effective therapeutic strategy against M2 macrophages in LSCC patients with lymph node metastasis.

## 1. Introduction

Despite advances being made in the detection and treatment of laryngeal squamous cell carcinoma (LSCC), the 5-year survival rate has not improved, particularly in patients who suffer from lymph node metastases [1]. Lymphangiogenesis is important in the pathogenesis of lymph node metastasis. Thus, new therapies to block the formation of new lymphatic vessels are urgently required.

HMGB1 is a nuclear protein involved in crucial biological processes [2]. Increased amounts of HMGB1 in tissues have been closely associated with the proliferation, invasion, metastasis, and prognosis of many tumors [3, 4]. More significantly, HMGB1 has been described as a damage-associated molecular pattern (DAMP) molecule in a number of infectious diseases and cancer [5, 6]. After being released from inflammatory cells, necrotic cells, or tumor cells, extracellular HMGB1 can bind to pattern recognition receptors [2, 7] and induce inflammation or promote tumor progression. The receptor of advanced glycation end products (RAGE) is a multiligand cell-surface receptor overexpressed in inflammation, cancer, and atherosclerosis [8–10]. Furthermore, RAGE is the major receptor for HMGB1 on macrophages [11]. RAGE has a greater affinity than Toll-like receptor (TLR) for HMGB1, although the HMGB1 pathway is also mediated by TLR2 and TLR4, and the cooperation between RAGE and TLRs has also been reported [7]. The HMGB1/RAGE signaling pathway regulates chemokines, cytokines, and adhesion molecules, which ultimately regulate cell proliferation, differentiation, and migration [12–14].

Macrophages are generated from monocytes, which have remarkable plasticity that allows them to efficiently respond to environmental signals and alter their phenotype. One phenotype is M2 macrophages, which when present in the tumor microenvironment can promote tumor cell proliferation, invasion, metastasis, and carcinogenesis. Classical M2 macrophages express CD163, CD206, and RAGE [15, 16]. Increasing evidence has suggested that HMGB1 and M2 macrophages are involved in lymph node metastasis [7].

Compelling evidence has indicated that the contribution of RAGE to tumor biology is not only its expression on cancer cells but also its specific enhancement of the inflammatory milieu in the tumor microenvironment. Although RAGE protein as a tumor cell receptor has been investigated [10, 17], the contribution of HMGB1/RAGE signaling to tumor lymphangiogenesis on M2 macrophages has not yet been investigated, at least to the best of our knowledge.

## 2. Materials and Methods

### 2.1. Patients and Tissue Preparation

A total of 280 paraffin-embedded samples were selected from the Department of Pathology, Beijing Tongren Hospital, Capital Medical University, between November 2009 and June 2011. Three experienced pathologists simultaneously confirmed the diagnosis and graded the hematoxylin-eosin-stained sections when neoplastic according to the 2017 4th Edition of the World Health Organization Classification of Head and Neck Tumors [18]. None of the patients had been treated with radiotherapy or chemotherapy. This study was approved by the Ethics Committee of Beijing Tongren Hospital of Capital Medical University, Beijing, China. As these samples were from samples obtained in the past, exemption from patient consent was obtained by the same ethics committee. The demographic and clinicopathological characteristics of the 280 patients are presented in Table 1. In total, 265 of the cases were male and 15 females, with a mean age of 59.8 years (range, 29-84 years). 101 of all patients had drunk alcohol; smoking index (which was the number of cigarettes per day multiplied by years of smoking) <400 was found in 166 patients. Smoking index ≥ 400 was found in 114 patients. A total of 55 patients had vocal polyp (VP), 102 had laryngeal precursor lesions (LPLs), 51 with low-grade dysplasia and 51 with high-grade dysplasia, and 123 had LSCC. Among the 123 LSCC cases, 10 were TNM stage I, 15 stage II, 55 stage III, and 43 stage IV. Lymph node metastasis was found in 57 cases. Vocal polyp (55) and LPLs (102) do not have TNM stage, lymph node metastasis, and differentiation. Seventy-eight patients died of laryngeal carcinoma. The 5­year survival rate was 63.4%. The overall survival was determined from the time of diagnosis to September 2019.

### 2.2. Immunohistochemistry

The slides were deparaffinized and dehydrated with descending grades of alcohol wash. Endogenous peroxidase activity was blocked, and antigen retrieval was conducted for 2.5 min in 0.01 mol/l (pH 6.0) trisodium citrate buffer. The slides were incubated with primary antibody to HMGB1 (ab18256, diluted 1 : 1.000), D2-40 (ab77854, diluted 1 : 40), and CD163 (ab182422, prediluted) (all from Abcam, No. 1 Kendall Square, Suite B2304 Cambridge, MA 02139-1517, USA) in an incubator at 37°C for 30 min. After being rinsed with phosphate-buffered saline, the slides were incubated with biotinylated goat anti-mouse/rabbit secondary antibody (ab6789/ab6721, diluted 1 : 1.000, Abcam) at 37°C for 15 min and then with streptavidin-biotin-peroxidase complex (ZSGB-BIO, Beijing, China). Slides processed with phosphate-buffered saline in place of the primary antibody were used as a negative control and esophageal squamous cell carcinoma as a positive control.

All immunohistochemistry slides were evaluated in a double-blinded manner. The HMGB1 expression was assessed in tumor cells with semiquantitative scoring: The staining distribution was scored as follows: 0, 0%; 1, 1-25%; 2, 26-50%; 3, 51-75%; and 4, 76-100%. The staining intensity was scored as follows: 0, negative; 1, weak; 2, medium; and 3, strong. The combined total staining scores ranged from 0 to 7, as previously described [19]. For statistical analysis, the HMGB1 expression was divided into a low expression (score of 0-4) and a high expression (score of 5-7).

To evaluate the density of CD163^+^ M2 macrophages, we scanned each specimen under low magnification (×40 and ×100) and selected 10 M2 macrophage-rich areas. CD163^+^ M2 macrophages were counted under magnification (×200) in the 10 areas, and the mean value of CD163^+^ M2 macrophages counted under magnification (×200) in the 10 areas represented the CD163^+^ M2 macrophage density for statistical analysis [20].

According to the published criteria [21], D2-40-positive staining (single endothelial cell or cell clusters) was interpreted as evidence of a lymphatic vessel. The vascular-rich area in peritumoral, intratumoral, and normal tissue was defined, and 10 fields of highly D2-40-positive vessels (hotspots) were counted under a magnification of ×200. The mean value of 10 fields of highly D2-40-positive vessels was calculated as the lymphatic vessel density (LVD). Taking the mean LVD value (5.42 ± 2.49 per 200 fields) as the cut-off, the cases were divided into high LVD and low LVD cases for statistical analysis.

### 2.3. Cell Culture

The human promonocytic cell line, THP-1, was obtained from the Cell Resource Center, Peking Union Medical College (Beijing, China). The human dermal lymphatic endothelial cells (HDLEC; Cat. no. C-12217) were purchased from PromoCell GmbH, Heidelberg, Germany.

The THP-1 cells were maintained in RPMI-1640 medium containing 10% fetal bovine serum (Nano Science and Technology Institute, Beijing, China), 100 units/ml penicillin, and 100 *μ*g/ml streptomycin (Sigma, St. Louis, MO 63103, USA). The HDLEC were cultured in endothelial cell basal medium MV2 (ECBM; Cat. no. C-22221, PromoCell), supplemented with 100 units/ml penicillin and 100 *μ*g/ml streptomycin. All the cells were incubated at 37°C in a humidified 5% CO_2_ atmosphere.

### 2.4. Stable RNA Transfection

The RAGE expression in the THP-1 cells has previously been documented [15]. To obtain a RAGE-THP-1 cell line, the transfection of RAGE shRNA lentiviral particles was used to knock down RAGE gene expression. In brief, the THP-1 cells were resuspended in RPMI-1640 medium with 10% FBS. A total of 1 × 10^3^ THP-1 cells were transfected with RAGE shRNA lentiviral particles (sc-36374-V, Santa Cruz Biotechnology, Inc., 10410 Finnell Street Dallas, Texas 75220, USA), the nontargeting control sequence (sc-108080), or copGFP control plasmid (sc-108084) in 96-well plates, as recommended by the manufacturer (Santa Cruz Biotechnology, Inc., 10410 Finnell Street Dallas, Texas 75220, USA). Following 1.5 months of puromycin selection, stable cultures of THP-1 cells with RAGE-targeted knockdown were selected and cloned for the induction of macrophages. The efficiency of RAGE-targeted knockdown in the macrophages was examined by western blot analysis.

### 2.5. Polarization of THP-1-Derived Macrophages and Collection of Conditioned Medium

Macrophages were obtained by the phorbol 12-myristate 13-acetate- (PMA-) induced differentiation of THP-1 cells. To achieve RAGE^+/-^ M0-polarized macrophages, 1 × 10^6^ RAGE^+/-^ THP-1 cells were incubated with 10 ng/ml PMA for 24 h. RAGE^+/-^ M2 macrophages were generated by stimulating the RAGE^+/-^ THP-1 cells with 10 ng/ml PMA for 6 h followed by incubation with PMA plus 20 ng/ml interleukin (IL)-13 and 20 ng/ml IL-4 (PeproTech, China, Room 416, 2 Diamond Plaza, 99 Yushan Road, Suzhou, Jiangsu, China) for 18 h, as previously described [22]. To remove differentiation stimuli, the macrophages were washed and incubated in the presence or absence of the RAGE ligand, HMGB1 (2 *μ*g/ml) (PeproTech, China, Room 416, 2 Diamond Plaza, 99 Yushan Road, Suzhou, Jiangsu, China), and anti-Toll-like receptor (TLR)2 (ab 91000, 1 *μ*g/ml) and anti-TLR4 (ab22048, 1 *μ*g/ml) antibodies (all from Abcam, No. 1 Kendall Square, Suite B2304 Cambridge, MA 02139-1517, USA) for 12 h. The collected conditioned media for use in subsequent experiments were the supernatants of RAGE^+/-^ M0 macrophages preconditioned with HMGB1 (2 *μ*g/ml), RAGE^+/-^ M0 macrophages preconditioned with HMGB1 (2 *μ*g/ml) and anti-TLR2 antibody (1 *μ*g/ml), RAGE^+/-^ M0 macrophages preconditioned with HMGB1 (2 *μ*g/ml) and anti-TLR4 antibody (1 *μ*g/ml), RAGE^+/-^ M2 macrophages preconditioned with HMGB1 (2 *μ*g/ml), RAGE^+/-^ M2 macrophages preconditioned with HMGB1 (2 *μ*g/ml) and anti-TLR2 antibody (1 *μ*g/ml), RAGE^+/-^ M2 macrophages preconditioned with HMGB1 (2 *μ*g/ml) and anti-TLR4 (1 *μ*g/ml), M0 macrophages, or M2 macrophages. ECBM alone was used as a control.

### 2.6. Western Blot Analysis

To evaluate whether classical M2 macrophages were obtained, CD206, CD163, and RAGE were evaluated by western blot. Total protein was obtained with radioimmunoprecipitation assay (RIPA) lysis buffer (Merck Millipore, 18/F, Building A, Phoenix Place, No. A5 Shuguangxili, Chaoyang District, Beijing, China) and quantified with the micro-BCA Protein Assay kit (Pierce Protein Biology/Thermo Fisher Scientific, Waltham, MA, USA). Western blot analysis was performed as previously described by Mahmood and Yang: 10 *μ*l protein loaded per lane, 8% SDS-PAGE gel, polyvinylidene difluoride (PVDF, KeyGen BioTECH, No. 207 room, Feng Bei Road, Fengtai District, Beijing, China), and 5% skimmed milk at 25°C in 60 minutes for blocking [23]. The primary antibodies (CD163, 1 : 1,000, ab87099; CD206, 1 : 1,000, ab64693; RAGE, 1 : 500, ab3611; and GAPDH, 1 : 1,000, ab22555, all from Abcam, No. 1 Kendall Square, Suite B2304 Cambridge, MA 02139-1517, USA) were incubated with the membranes overnight at 4°C. The membranes were then incubated with goat anti-rabbit/mouse IgG-HRP (1 : 5,000) at room temperature for 2 h and developed with the ECL system (Pierce Protein Biology/Thermo Fisher Scientific). ImageJ software was used to analyze the gray value of the bands.

### 2.7. Detection of mRNA Expression

To evaluate whether classical M2 macrophages were obtained, IL-1*β*, IL-12, IL-10, and CCL-13 were evaluated by RT-qPCR. Total RNA was extracted with the use of RNeasy mini kit (Cat. no. 74106, Qiagen, 19300 Germantown Road Germantown MD 20874 Valencia, CA, USA). To detect the transcript levels of IL-1*β*, IL-12, IL-10, and CCL-13, reverse transcription-PCR (RT-PCR) was performed using the PrimeScript® RT reagent kit with gDNA Eraser (Code No. RR047, Takara, Dalian, China), according to the manufacturer's instructions which include the following gDNA Eraser: 1 *μ*l; 5X gDNA Eraser Buffer: 2 *μ*l; RNA (1 *μ*g/*μ*l): 1 *μ*l; RNase Free dH_2_O: 6 *μ*l; and total liquid: 10 *μ*l were performed at 42°C in 2 minutes. Above reaction liquid: 10 *μ*l; 5 × PrimeScript Buffer 2: 4 *μ*l; PrimeScript RT Enzyme Mix I: 1 *μ*l; RT primer mix: 1 *μ*l; RNase Free dH_2_O: 4 *μ*l; and total liquid: 20 *μ*l were performed at 37°C in 15 minutes and 85°C in 5 seconds. Real-time PCR was performed using the SYBR Premix Ex Taq II (Code No. RR820, Takara, Dalian, China), according to the manufacturer's instructions which include the following: SYBR Premix Ex Taq II (Tli RNaseH Plus) (2x): 12.5 *μ*l; primer forward (10 *μ*M): 1 *μ*l; primer reverse (10 *μ*M): 1 *μ*l; cDNA: 2 *μ*l; dH2O: 8.5 *μ*l; and total liquid: 25 *μ*l were performed at 95°C in 30 seconds, then 95°C in 5 seconds, and 60°C in 30 seconds at 45 cycles and with GAPDH as a control gene. The primers used were as follows: GAPDH forward, 5′-GAAGGTGAAGGTCGGAGT-3′ and reverse, 5′-GAAGATAATGATGGGATTTC-3′; IL-1*β* forward, 5′-AGTGCCTTGAGATTCT-3′ and reverse, 5′-GGTATGCCACTATGCAT-3′; IL-10 forward, 5′-AGTGGGGATGTTAGCCCT-3′ and reverse, 5′-TAGGTTCTCTGGAATTG-3′; IL-12 forward, 5′-AGTGGAGTGCCAGGAGGACA-3′ and reverse, 5′-TTCTTGGGTGGGTCAGGTTT-3′; and CCL-13 forward, 5′-GCTGACCCAAAGGAGAAGTG-3′ and reverse, 5′-CCAAAGCATAGAAGAGGAGGC-3′.

Lymphangiogenesis is a major biological function of M2 macrophages in promoting tumor progression. The process is described as HDLEC proliferation, migration, and preservation of lymphatic vessels.

### 2.8. HDLEC Proliferation Assay

HDLEC proliferation was determined using the Cell Counting Kit-8 (CCK-8; Beyotime, Beijing, China) assay. A total of 2 × 10^3^ HDLEC were seeded in 96-well plates with 200 *μ*l ECBM. The medium was removed following incubation for 6 h, and the HDLEC were then incubated, respectively, with 200 *μ*l collection-conditioned medium from the supernatants of the RAGE^+/-^ M0 macrophages preconditioned with HMGB1 (2 *μ*g/ml), RAGE^+/-^ M0 macrophages preconditioned with HMGB1 (2 *μ*g/ml) and anti-TLR2 antibody (1 *μ*g/ml), RAGE^+/-^ M0 macrophages preconditioned with HMGB1 (2 *μ*g/ml) and anti-TLR4 antibody (1 *μ*g/ml), RAGE^+/-^ M0 macrophages preconditioned with HMGB1 (2 *μ*g/ml) and anti-vascular endothelial growth factor (VEGF)-C antibody (1 ng/ml,ab9546, Abcam, No. 1 Kendall Square, Suite B2304 Cambridge, MA 02139-1517, USA), RAGE^+/-^ M2 macrophages preconditioned with HMGB1 (2 *μ*g/ml) and anti-VEGF-C antibody (1 ng/ml), RAGE^+/-^ M2 macrophages preconditioned with HMGB1 (2 *μ*g/ml), RAGE^+/-^ M2 macrophages preconditioned with HMGB1 (2 *μ*g/ml) and anti-TLR2 antibody (1 *μ*g/ml), RAGE^+/-^ M2 macrophages preconditioned with HMGB1 (2 *μ*g/ml) and anti-TLR4 antibody (1 *μ*g/ml), or M0 macrophages or M2 macrophages. ECBM (Endothelial Cell Base Medium MV2) alone was used as a control. All media were removed after 48 h, and the HDLEC were cultivated with 20 *μ*l CCK-8 in each well at 37°C for 30 min. The absorbance was measured at 450 nm. The experiments were performed in triplicate.

### 2.9. HDLEC Migration Assay

The HDLEC migration assay was performed with 24-well polycarbonate Transwell (8 *μ*m pore sizes). A total of 5 × 10^4^ HDLEC were resuspended in 200 *μ*l ECBM and added to the upper compartments. Collection-conditioned medium from the supernatants was added to the lower chambers, respectively. ECBM alone was used as a control. Six hours later, migrating HDLEC were fixed and stained with crystal violet at 25°C in 5 minutes (C0121, Beyotime Biotechnology, No. 30, Xinfei Road, Songjiang District, Shanghai, China). HDLEC on the upper surface of the filter membrane were removed by a cotton swab. The migrating HDLEC were counted in 10 fields at ×200 magnification. The experiments were performed in triplicate. Data are presented the means ± SD of 3 independent experiments.

### 2.10. Lymphangiogenesis Assay

For this assay, 96-well plates were coated with 50 *μ*l Matrigel for 30 min at 37°C, and 1 × 10^4^ HDLEC in 50 *μ*l ECBM were seeded on the plates. Subsequently, 150 *μ*l of collection-conditioned medium from the supernatants or ECBM was added, respectively, to cell cultures and incubated for 6 h at 37°C. Lymphangiogenesis was quantified by counting the number of tube-like structures in 10 fields at ×100 magnification using ImageJ software. Each experiment was repeated 3 times as previously described [24].

### 2.11. ELISA for Supernatants and Conditioned Medium

The concentrations of tumor necrosis factor (TNF)-*α*, transforming growth factor (TGF)-*β*, IL-10, and IL-23 in the supernatants of M0 and M2 macrophage cultures were measured using commercial ELISA kits (R&D Systems, Minneapolis, MN, USA) according to the manufacturer's instructions. The levels of VEGF-C in 6 different conditioned media and ECBM were also measured.

### 2.12. Statistical Analysis

Quantitative data are presented as the means ± SD. Significant differences between the 2 groups were analyzed using a two-sided Student's *t*-test, and differences between multiple groups were analyzed with a two-sided ANOVA with Dunnett's post hoc test. For the overall survival rate, the Kaplan-Meier test with the log rank test was used. The correlation analysis was performed using Spearman's correlation analysis. The data of the HMGB1 expression and CD163^+^ M2 macrophage density were analyzed using the *χ*^2^ test and Student's *t*-test, respectively (except for lesion variables). The data of lesion variables were analyzed using ANOVA with Dunnett's post hoc test. Qualitative data were representative of three independent experiments. All analyses were conducted using SPSS20 software. A *P* value < 0.05 was considered to indicate a statistically significant difference.

## 3. Results

### 3.1. HMGB1 and CD163+ M2 Macrophage Density in Laryngeal Vocal Polyp, Laryngeal Precursor Lesions, and LSCC

**Figure 1:**
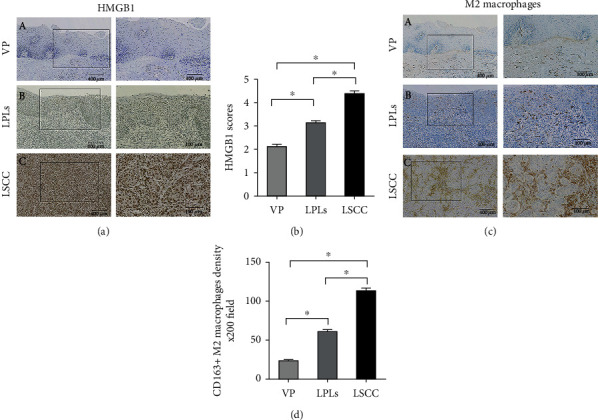
Immunohistochemical staining and semiquantitative evaluation of HMGB1 and CD163. (a) HMGB1 expression is scored as 2 in VP (a-A), 4 in LPLs (a-B), and 6 in LSCC (a-C) specimens. Original magnification, ×40 (left column), and selected areas (boxed areas), ×10. Semiquantitative evaluation of (b) HMGB1. To identify M2 macrophages in the tissue samples, an anti-CD163 antibody was used. (c) M2 macrophage density was greater in LSCCs (c-C) and LPLs (c-B) than in VP(c-A). Original magnification, ×40 (left column), and selected areas (boxed areas), ×10. Semiquantitative evaluation of (d) M2 macrophage density. Asterisks indicate statistically significant differences (^∗^*P* < 0.05).

Firstly, using immunohistochemistry, the HMGB1 expression was examined in the VP, LPLs, and LSCC (Figure 1). HMGB1 was mainly detected in the nuclei of the tumor cells, nontumor epithelial cells, and inflammatory cells. The HMGB1 expression was lowest in the VP (Figure 1(a)-A)), highest in LSCC (Figure 1(a)-C)), and intermediate in the LPLs (Figure 1(a)-B)).  Figure 1(b) also illustrates that the HMBG1 expression was higher in the LSCC than in the LPLs (*P* < 0.05) and VP (*P* < 0.05).

M2 macrophages express CD163 in their cytoplasm, whereas tumor cells do not. In this study, CD163^+^ M2 macrophages were mostly infiltrated in the tumor stroma, and the cells were greater in number in the LPLs (Figure 1(c)-B)) and LSCC (Figure 1(c)-C)) than in the VP (Figure 1(c)-A)). Similarly, the CD163^+^ M2 macrophage density was significantly higher in the LPLs (*P* < 0.05) and LSCC (*P* < 0.05) than in the VP (*P* < 0.05) (Figure 1(d)).

### 3.2. Association between the Patient Clinicopathological Characteristics and HMGB1 Expression and CD163+ M2 Macrophage Density

**Figure 2:**
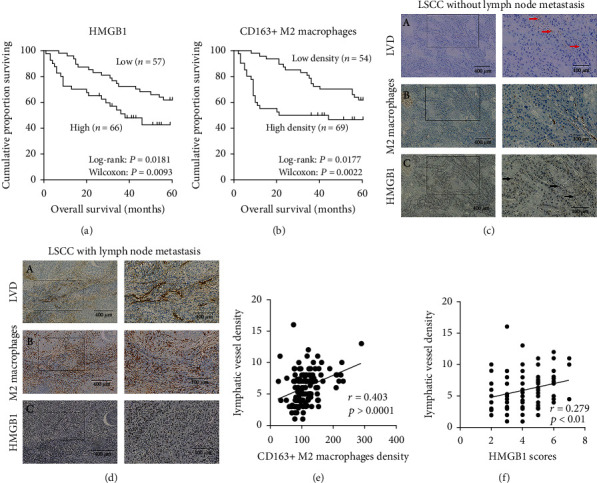
Association between the survival of patient and HMGB1 and CD163+ M2 macrophage density. The Kaplan-Meier analysis of overall survival in patients based on (a) HMGB1 and (b) M2 macrophage density. To identify LVD, a D2-40 antibody was used. (c) LVD, (red arrow (c-A)), M2 macrophage density (c-B), and HMGB1 (black arrow (c-C)) were lower in LSCC without lymph node metastasis than (d) with lymph node metastasis. (e) A positive correlation between the CD163+ M2 macrophage density and LVD is illustrated. (f) A positive correlation between HMGB1 and LVD is illustrated.

The HMGB1 expression and clinicopathological variables are presented in Table 2. A high HMGB1 expression was significantly associated with an advanced stage (stages III and IV; *P* < 0.001) and lymph node metastasis (*P* < 0.001). Among the LSCC samples with lymph node metastasis, the HMGB1 expression was high in 41 samples (33.4%). Survival was poorer in patients with a high HMGB1 expression in LSCC than in those with a low HMGB1 expression (*P* < 0.05, Figure 2(a)). On the whole, the results indicated that HMGB1 was overexpressed in the majority of patients and was associated with lymph node metastasis and a poor prognosis.

The associations between the clinicopathological variables with CD163^+^ M2 macrophage density are also presented in Table 2. A high density of CD163^+^ M2 macrophage infiltration was significantly associated with lymph node metastasis (*P* = 0.006). In the LSCC samples with lymph node metastasis, the mean density of CD163^+^ M2 macrophage infiltration was 124.2 ± 52.7 per 200 fields, whereas in the LSCC samples without lymph node metastasis, the mean density of CD163^+^ M2 macrophage infiltration was 103.3 ± 28.1 per 200 fields. Moreover, the prognosis for patients with a low density of CD163^+^ M2 macrophage infiltration in LSCC was more favorable than that of patients with a high density of CD163^+^ M2 macrophage infiltration (*P* < 0.05, Figure 2(b)).

### 3.3. HMGB1 Expression and CD163+ M2 Macrophage Density Are Significantly Associated with Lymphatic Vessel Density in LSCC

Based on the above-mentioned data, we sought to determine whether the HMGB1 expression and M2 macrophage density are involved in lymphangiogenesis in LSCC. The association between HMGB1 expression, the mean density of CD163^+^ M2 macrophages, and the quantity of D2-40 positive lymphatic vessels were analyzed. The mean LVD was 5.42 ± 2.49 per 200 fields in LSCC; <5.42 per 200 fields were considered low LVD (Figure 2(c)) and ≥5.42 per 200 fields were considered high LVD (Figure 2(d)). The HMGB1 overexpression was observed in 38.2% of the LSCC samples with a high LVD compared with 15.5% with a low LVD (*P* < 0.001, Table 2). The mean CD163^+^ M2 macrophage density was 111.4 ± 30.1 and 91.4 ± 19.4, respectively, in the samples with a high and low LVD (*P* = 0.002, Table 2). CD163^+^ M2 macrophage density and LVD positively correlated (*r* = 0.403, *P* < 0.0001, Figure 2(e)). Similarly, the HMGB1 expression positively correlated with LVD (*r* = 0.279, *P* < 0.01, Figure 2(f)). These data suggest that an increased expression of HMGB1 and a high density CD163^+^ M2 macrophages promote lymphangiogenesis in LSCC.

### 3.4. Classic Markers of M2 Macrophages and M0 Macrophages

The RAGE expression in the THP-1 cells has previously been documented [15] and was confirmed in the present study. Therefore, we also used RAGE shRNA lentiviral particle transfection to knock down RAGE gene expression and generated a RAGE^+/-^ THP-1 cell line, which was used for macrophage differentiation. We achieved M0 macrophages and M2 macrophages by the use of a previously reported protocol [22].

**Figure 3:**
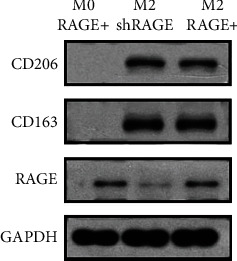
Classic protein markers of M2 macrophages (CD206 and CD163) assessed by western blot analysis. CD206 and CD163 are equally expressed in RAGE^+^ and RAGE^−^ M2 macrophages in which RAGE was knocked down (M2 macrophage shRAGE), but they are negative in M0 macrophages. The RAGE expression was lower in M2 macrophage shRAGE than in RAGE^+^ M2 macrophages and M0 macrophages.

**Figure 4:**
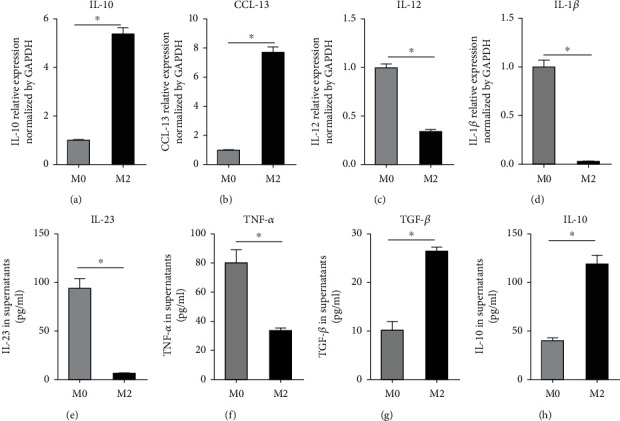
Classic cytokine and chemokine expressions in M2 macrophages and M0 macrophages. RT-qPCR analysis indicated that (a) IL-10 and (b) CCL-13 mRNA expressions were high in M2 macrophages. M0 macrophages expressed higher levels of (c) IL-12 and (d) IL-1*β* mRNA than M2 macrophages. The distribution of these markers is a classic pattern. Additionally, the lower levels of (e) IL-23 and (f) TNF-*α* protein and the higher levels of (g) TGF-*β* and (h) IL-10 protein were measured in the cell culture supernatants of M2 macrophages and M0 macrophages (^∗^*P* < 0.05).

The expression levels of IL-1*β*, IL-12, IL-10, and CCL-13 were evaluated by RT-qPCR. The protein levels of CD163, CD206, and RAGE were assessed with western blot analysis. CD163 and CD206 were strongly expressed in M2 macrophages; the expression levels of RAGE were low in the M2 macrophages in which RAGE was knocked down (M2 macrophage shRAGE) (Figure 3). The M0-differentiated macrophages expressed higher levels of IL-1*β* and IL-12 than did the differentiated M2 macrophages. Moreover, the M2 macrophages exhibited a classic pattern as regards the levels of IL-23, TNF-*α*, TGF-*β*, and IL-10 measured in the cell culture supernatants (Figure 4). Thus, we successfully obtained classical M2 macrophages and M0 macrophages.

### 3.5. HMGB1 Promotes HDLEC Proliferation, Migration, and Lymphangiogenesis by the Activation of RAGE on M2 Macrophages In Vitro

**Figure 5:**
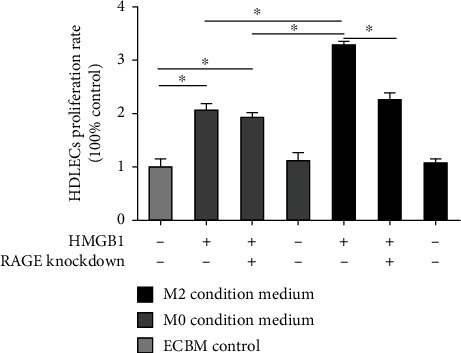
HDLEC incubated with conditioned medium from HMGB1-stimulated RAGE+ M2 macrophages had the highest rate of proliferation. Graphical illustration of statistical results of HDLEC proliferation rate of incubation with different conditioned media by CCK8 proliferation assay. Multiple comparisons were performed by ANOVA with the Dunnett's post hoc test. Data are the means ± SD of 3 independent experiments (^∗^*P* < 0.05).

Lymphangiogenesis is a major biological function of M2 macrophages in promoting tumor progression. The process is described as lymphatic endothelial cell proliferation, migration and remodeling, and preservation of lymphatic vessels. Thus, in order to assess whether the HMGB1-induced activation of RAGE on CD163^+^ M2 macrophages promotes lymphangiogenesis, we evaluated HDLEC proliferation with CCK-8 assay after incubation with 6 different conditioned media or ECBM (control) in vitro. We observed that HDLEC incubated with conditioned medium from HMGB1-stimulated RAGE^+^ M2 macrophages had the highest proliferation ability (*P* < 0.05). In addition, when the HDLEC were cultured with conditioned medium from M0 macrophages treated with HMGB1, a greater proliferation of HDLEC was observed compared with that of the controls (*P* < 0.05). RAGE knockdown decreased the proproliferative effects of HMGB1 preconditioned M2 macrophages on HDLEC (*P* < 0.05, Figure 5).

**Figure 6:**
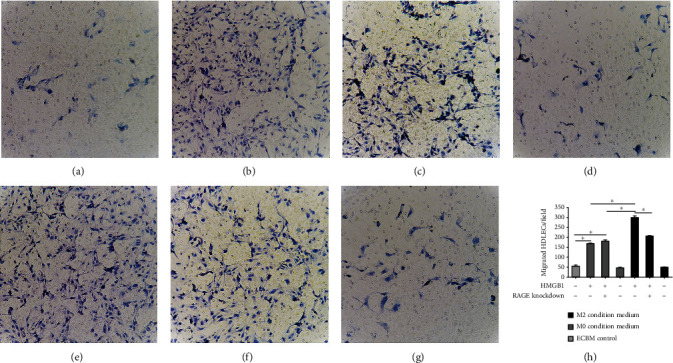
The activation of RAGE^+^ M2 macrophages by HMGB1 can potentiate the HDLEC migratory activity. Migrating HDLEC, stained with crystal violet, were counted in 10 fields (×200 magnification) after incubated with conditioned medium of (a) ECBM (control), (b) RAGE^+^ M0 macrophages preconditioned with HMGB1, (c) RAGE-targeted knockdown in M0 macrophages treated with HMGB1, (d) M0 macrophages alone, (e) RAGE^+^ M2 macrophages preconditioned with HMGB1, (f) RAGE-targeted knockdown in M2 macrophages treated with HMGB1, or (g) M2 macrophages alone. (h) Graphical illustration of statistical results of migrating HDLEC of incubation with different conditioned media by Transwell migration assay. Multiple comparisons were performed by ANOVA with the Dunnett's post hoc test. Data are the means ± SD of 3 independent experiments (^∗^*P* < 0.05).

To determine whether the activation of RAGE^+^ M2 macrophages by HMGB1 can potentiate the HDLEC migratory activity, we performed a cell migration assay. The results revealed that conditioned medium from the RAGE^+^ M2 macrophages treated with HMGB1 significantly promoted HDLEC migration in comparison with that of the other conditioned media (*P* < 0.05; Figure 6); however, RAGE-targeted knockdown in M2 macrophages treated with HMGB1 markedly reduced the promigratory effects of the M2 macrophage-conditioned medium (*P* < 0.05; Figure 6). Moreover, conditioned medium from M0 macrophages stimulated with HMGB1 also enhanced the HDLEC migratory activity compared with that of the control (*P* < 0.05, Figure 6).

**Figure 7:**
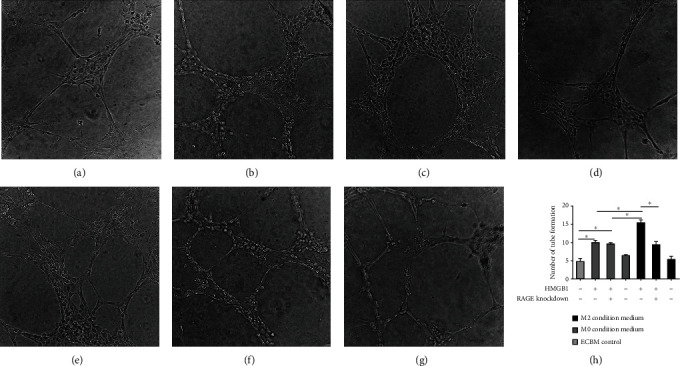
Conditioned medium from M0 macrophages preconditioned with HMGB1 also increased lymphangiogenesis. The number of lymphangiogenesis was counted in 10 fields (×100 magnification) after incubated with conditioned medium of (a) ECBM (control), (b) RAGE^+^ M0 macrophages preconditioned with HMGB1, (c) RAGE-targeted knockdown in M0 macrophages treated with HMGB1, (d) M0 macrophages alone, (e) RAGE^+^ M2 macrophages preconditioned with HMGB1, (f) RAGE-targeted knockdown in M2 macrophages treated with HMGB1, or (g) M2 macrophages alone. (H) Graphical illustration of statistical results of lymphangiogenesis number after incubation with different conditioned media by lymphangiogenesis assay. Multiple comparisons were performed by ANOVA with Dunnett's post hoc test. Data are the means ± SD of 3 independent experiments (^∗^*P* < 0.05).

To evaluate lymphangiogenesis, 6 different media or the ECBM control were added to HDLEC placed in Matrigel-coated wells. Lymphangiogenesis was significantly increased when the HDLEC were incubated with medium from RAGE^+^ M2 macrophages treated with HMGB1 (*P* < 0.05; Figure 7); RAGE knockdown decreased the prolymphangiogenic effects of HMGB1-preconditioned M2 macrophages on the HDLEC (*P* < 0.05; Figure 7). In addition, conditioned medium from M0 macrophages preconditioned with HMGB1 led to an increase in the lymphatic vessel network compared with that of the control (*P* < 0.05, Figure 7).

**Figure 8:**
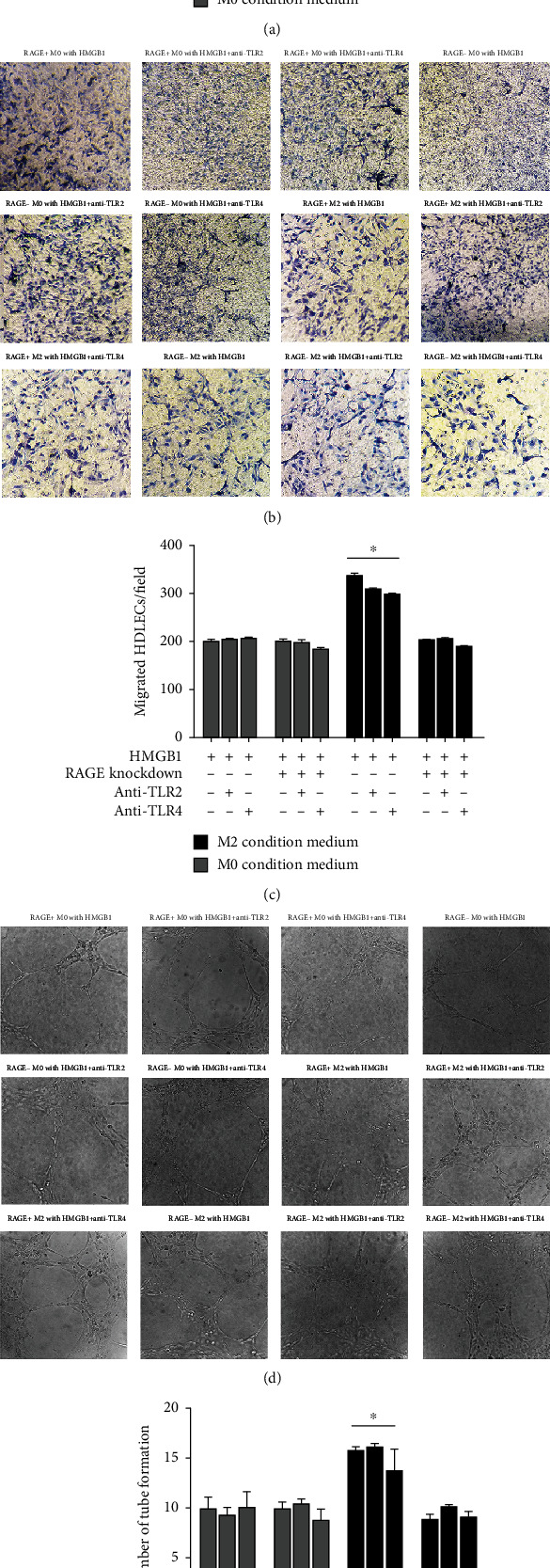
HMGB1-TLR pathway does not significantly increase HDLEC proliferation, migration, and lymphangiogenesis on M2 macrophages. HDLEC incubated, respectively, with conditioned medium from RAGE^+/-^ M0 macrophages preconditioned with HMGB1, RAGE^+/-^ M0 macrophages preconditioned with HMGB1 and anti-TLR2/anti-TLR4 antibody, RAGE^+/-^ M2 macrophages preconditioned with HMGB1, or RAGE^+/-^ M2 macrophages preconditioned with HMGB1 and anti-TLR2/anti-TLR4 antibody. (a) CCK-8 assays were performed to assess the proliferation of HDLEC under various treatment conditions. HDLEC were seeded into upper chamber of Transwell plates and counted by light microscopy after 6 h. Representative (b) micrographs and (c) cell counts for migration are shown. The formation of lymphatic vessels was counted in Matrigel after 6 h. Representative (d) micrographs and (e) formation of lymphatic vessels counts are shown. Data are presented as the means ± SD and are representative of 3 independent experiments (^∗^*P* < 0.05).

The importance of the HMGB1-induced activation of RAGE on M2 macrophages was further confirmed by treating the M2 macrophages with anti-TLR2 antibody and anti-TLR4 antibody, none of which inhibited the proliferative ability of the HDLEC; the HMGB1-TLR pathway did not significantly increase HDLEC migration on M2 macrophages. Similar results were observed with the migratory ability and lymphangiogenesis (Figure 8).

### 3.6. VEGF-C in Collection of Conditioned Medium

**Figure 9:**
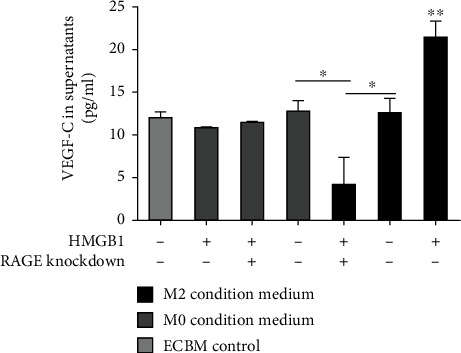
HMGB1-induced VEGF-C production is significantly higher in conditioned medium from RAGE+ M2 macrophages. VEGF-C concentration in different conditioned medium was measured by ELISA. Multiple comparisons were performed by ANOVA with Dunnett's post hoc test. Data are the means ± SD of 3 independent experiments (^∗^*P* < 0.05 and ^∗∗^*P* < 0.01).

Cytokines are important in the functionality and phenotypic polarization of macrophages. VEGF-C is a major cytokine involved in the lymphangiogenesis. Thus, in this study, we examined the VEGF-C concentration in 6 different conditioned media and ECBM (control). We found that HMGB1-induced VEGF-C production was significantly higher in RAGE^+^ M2 macrophage-conditioned medium (*P* < 0.01), and RAGE knockdown decreased the VEGF-C concentration in the conditioned medium from HMGB1 preconditioned M2 macrophages (*P* < 0.05, Figure 9). Of note, the VEGF-C level in the conditioned medium from M0 macrophages stimulated with HMGB1 was not increased compared with that from the control (*P* >0.05).

**Figure 10:**
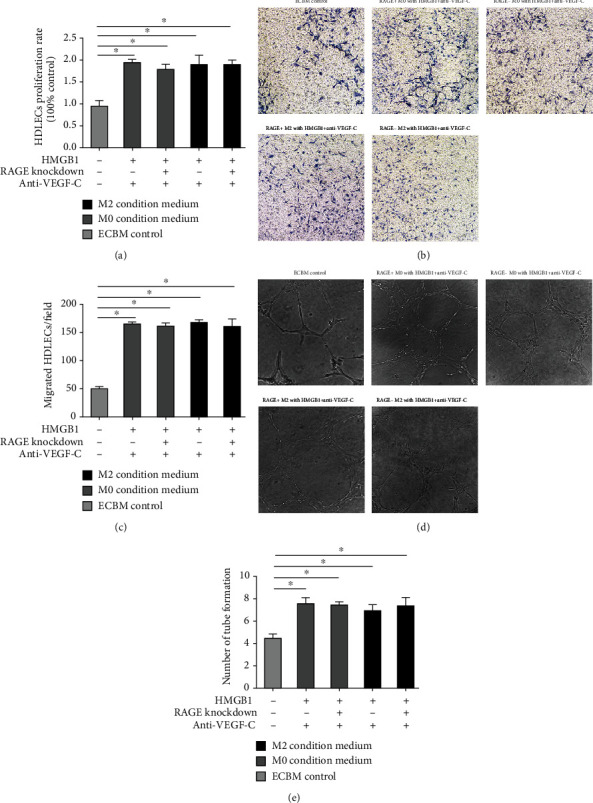
Anti-VEGF-C antibody decreases the proliferation, migration, and lymphangiogenesis of HDLEC cultured with RAGE^+^ M2 macrophages preconditioned with HMGB1. HDLEC were incubated, respectively, with conditioned medium from RAGE^+/-^ M0 macrophages preconditioned with HMGB1 and anti-VEGF-C antibody or RAGE^+/-^ M2 macrophages preconditioned with HMGB1 and anti-VEGF-C antibody or ECBM. (a) CCK-8 assays were performed to assess the proliferation of HDLEC under various treatment conditions. HDLEC were seeded into upper chamber of Transwell plates and counted by light microscopy. Representative (b) micrographs and (c) cell counts for migration are shown. The formation of lymphatic vessels of HDLEC was counted in Matrigel. Representative (d) micrographs and (e) formation of lymphatic vessels counts are shown. Data are presented as the means ± SD and are representative of 3 independent experiments (^∗^*P* < 0.05).

The importance of the VEGF-C induction after the activation of RAGE on M2 macrophages was further confirmed by the addition of anti-VEGF-C antibody to the conditioned medium. The results revealed that HDLEC incubated with conditioned medium from RAGE^+^ M2 macrophages, preconditioned with HMGB1 and anti-VEGF-C antibody, exhibited a proliferation ability similar to that of the HDLEC incubated with the other conditioned media (Figure 10(a)). Moreover, we observed that the RAGE-targeted knockdown in the M2 macrophages treated with HMGB1 and anti-VEGF-C antibody did not markedly reduce the promigratory effects of the M2 macrophage-conditioned medium (Figures 10(b) and 10(c)). Similar results were observed with lymphangiogenesis (Figures 10(d) and 10(e)). Of note, the HDLEC incubated with conditioned medium from M2 macrophages or M0 macrophages treated with HMGB1 exhibited a greater proliferative activity and lymphangiogenesis capacity compared with that of the controls (*P* < 0.05, Figures 10(a), 10(d), and 10(e)).

## 4. Discussion

For many patients with LSCC, early evidence of tumor spread is regional draining lymph node metastasis, which leads to the main cause of cancer-related mortality [1]. Over the past decade, the understanding of the complex molecular mechanisms involved in lymphangiogenesis has markedly improved [25, 26]; however, no antilymphangiogenic compounds have yet been approved for use in clinical practice, at least to the best of our knowledge. New or enhanced therapies to block the formation of new lymphatic vessels are urgently required.

An increased HMGB1 expression has been reported to be closely associated with the proliferation, invasion, metastasis, and prognosis of tumors [3, 4]. Tumor cells secrete cytokines to recruit monocytes infiltrated in the tumor stroma and promote their differentiation and polarization to M2 macrophages [27]. A compelling body of evidence has indicated the involvement of HMGB1 and M2 macrophages in lymphatic metastasis [28, 29].

In this study, in immunohistochemistry experiments, we found that HMGB1 and CD163^+^ M2 macrophage densities were increased with the development of the disease (from VP, LPL to LSCC) and that the HMBG1 expression was significantly higher in LSCC (*P* < 0.05). Other studies [30, 31] have made similar observations in other tumors: In the carcinogenesis of gastric and cervical tumors, the expression level of HMGB1 has been found to be increased in the sequence of epithelial metaplasia-dysplasia-cancer. In addition, HMGB1 protein overexpression has been shown to be involved in lymph node metastasis and to be associated with a poor prognosis of patients with LSCC [32, 33], as we have found. It has been previously reported [17] that HMGB1 interaction with RAGE activates the NF-*κ*B/STAT3 pathway, which are molecular effector mechanisms linked to tumor cell proliferation and invasion, in a mouse model of lung cancer, and the blockade of HMGB1 can be targeted to suppress tumor development and metastasis. The results of this study revealed that the density of CD163^+^ M2 macrophages was significantly higher in tissue samples from patients who had a poor prognosis (*P* < 0.05) or with lymph node metastasis (*P* < 0.05). Lin et al. [28] reported that increased CD163^+^ tumor-associated macrophage (TAM) infiltration in LSCC can be a marker of metastasis and prognosis, but the authors did not provide density counts of the infiltration. In this study, we provided the mean density values of CD163^+^ M2 macrophage infiltration, which is a more objective prediction of the prognosis of patients with LSCC.

In complementary experiments, we found that HMGB1 protein expression and CD163^+^ M2 macrophage infiltration were positively associated with LVD (*P* < 0.01). A previous study reported that plentiful lymphangiogenesis was observed in patients with pancreatic cancer with nodal metastasis [34], which was consistent with our findings. Moreover, Kurahara et al. [34] found that the higher LVD was associated with higher densities of CD163^+^ TAMs in pancreatic tumors, which also was similar to the present findings in LSCC (*P* < 0.05). Thus, we hypothesized that HMGB1 promotes lymphangiogenesis through the activation of RAGE on M2 macrophages and established an in vitro model to examine this hypothesis. We found that the treatment of RAGE^+^ M2 macrophages with HMGB1 promoted lymphangiogenesis by inducing HDLEC proliferation, migration, and vessel formation, as evidenced by the RAGE-targeted knockdown leading to decrease lymphangiogenesis (*P* < 0.05).

RAGE is a type I transmembrane protein, with carboxylation N-glycosylation, which promotes the binding of HMGB1 and signal transduction [7, 8]. RAGE has a greater affinity than TLR for HMGB1, although the HMGB1 pathway is also mediated by TLR2 and TLR4, and the cooperation between RAGE and TLRs has also been reported [7]. Cells of various types and the pathophysiological context seem to determine this partnership and determine which receptor is dominant [35]. Moreover, in this study, when TLR2 and TLR4 were blocked, the proliferative ability and lymphangiogenesis of HDLEC were not significantly inhibited, although RAGE knockdown decreased the effects of HMGB1 stimulation. Thus, this suggests that the HMGB1-TLR pathway is not crucial for lymphangiogenesis in HDLEC, as TLR2 needs to bind to HMGB1-containing nucleosomes to induce cytokine production, whereas HMGB1 alone does not [36]. Therefore, the contribution of RAGE seems to be predominant in HMGB1-induced prolymphangiogenesis. M2 macrophages produce a number of potent vascular growth factors, such as VEGF-C, which stimulate lymphatic vessel formation [37]. The secretion of VEGF-C by macrophages, in particular, seems to be important for proangiogenesis in tissue repair [38], and decreased VEGF-C secretion in granulation tissue is followed by significantly reduced angiogenesis [39]. The recruitment and abundant infiltration of macrophages and the secretion of VEGF-C stimulate lymphangiogenesis, and this has been proven by a number of studies [40, 41]. Suzuki et al. proposed that TGF-*β* upregulated VEGF-C expression in macrophages, thus enhancing lymphangiogenesis [42]. TNF receptor 1 activation in macrophages by TNF-*α* has been shown to promote the expression of VEGF-C, which in turn induces VEGFR3 on lymphatic endothelial cells [43]. The data of the present study on the HDLEC lymphangiogenesis assay revealed that RAGE^+^ M2 macrophages preconditioned with HMGB1 produced significant higher levels of VEGF-C, which could promote lymphangiogenesis (*P* < 0.05). However, anti-VEGF-C antibody decreased the prolymphangiogenic effects of HMGB1-preconditioned RAGE^+^ M2 macrophages on HDLEC. Thus, it is suggested that the secretion of VEGF-C is a key factor for the stimulation of lymphangiogenesis during the process of RAGE activation on M2 macrophages.

The results of this study also demonstrated that although HMGB1-treated RAGE^+^ M0 macrophages exhibited an increase in lymphangiogenesis, lymphangiogenesis was significant less than that of the HMGB1-treated RAGE^+^ M2 macrophages, even though RAGE was equally expressed in the M0 macrophages and M2 macrophages. As RAGE gene has extreme polymorphism, RAGE mRNA is one of the main reprogramming changes in the transcriptome during the macrophage polarization process [44, 45]. Polymorphisms may affect gene transcriptional activity and result in differences in binding affinity to ligands. Thus, RAGE on M2 macrophages may have a higher affinity for HMGB1 than do M0 macrophages. Although RAGE activation of classic proinflammatory responses has been studied extensively, the data from the present study illustrate that the macrophage status is important in altering RAGE traditional proinflammatory responses. Moreover, we used a reduced PMA concentration, which may also improve the response of macrophages to HMGB1 during THP-1 cell differentiation to macrophages [22]. The data of this study also revealed that HMGB1-treated M0 macrophages promoted HDLEC proliferation compared with control (*P* < 0.05), but not in parallel with increments in VEGF-C levels, and HDLEC proliferation did not differ between the cells in which RAGE was knocked down or not and treated with HMGB1 and anti-VEGF-C antibody on M0 macrophages and M2 macrophages. A possible reason for this may be the difference is the enhancing effects of HMGB1, a proangiogenic cytokine, on HDLEC proliferation, and HMGB1 alters endothelial cell function by activating the expression of intercellular adhesion molecule 1 and vascular cell adhesion molecule 1 [46]. However, the question of whether HMGB1 needs to cooperate with other molecules in lymphatic endothelial cells to mediate lymphangiogenesis remains unclear.

## 5. Conclusion

This study on LSCC revealed that HMGB1 increases prolymphangiogenic properties through the activation of RAGE on M2 macrophages, as evidenced by the RAGE-targeted knockdown, which decreased lymphangiogenesis. A HMGB1 expression and density of CD163^+^ M2 macrophages may provide clues to the mechanisms of lymphatic metastasis in LSCC and may lead to the development of novel therapeutic strategies which to target RAGE on M2 macrophages. Future endeavours are warranted to explore the downstream signaling pathways of RAGE on M2 macrophages in lymph node metastasis in LSCC.

## Figures and Tables

**Table 1:** Demographics and clinicopathological characteristics of all 280 cases.

Characteristics	Patient (*n*%)
Sex	
Male	265 (94.6%)
Female	15 (5.4%)
Age	
Mean (y)	59.8
Range (y)	29-84
<60 y	126 (45%)
≥60 y	154 (55%)
Lesion	
Vocal polyp	55 (19.6%)
LPLs	102 (36.5%)
LSCC	123 (43.9%)
Differentiation	
Well	28 (22.7%)
Moderate	72 (58.6%)
Poorly	23 (18.7%)
Smoking index	
<400	166 (59.3%)
≥400	114 (40.7%)
Drink habit	
None	179 (63.9%)
Drink	101 (36.1%)
TNM stage	
Stage I	10 (8.1%)
Stage II	15 (12.1%)
Stage III	55 (44.9%)
Stage IV	43 (34.9%)
Lymph nodes	
Negative	66 (53.7%)
Positive	57 (46.3%)

LPLs: laryngeal precursor lesions; LSCC: laryngeal squamous cell carcinoma.

**Table 2:** Correlation of clinicopathological variables with HMGB1 expression and CD163+ M2 macrophage density.

Variables	Patients *n*	Low HMGB1 *n* (%)	High HMGB1 *n* (%)		CD163+M2 macrophages	
		(scored 0–4)	(scored 5–7)	*P*	(Mean ± SD)	*P*
Sex				0.60		0.16
Male	265	196 (70)	69 (24.6)		77.4 ± 45.7	
Female	15	12 (4.3)	3 (1.1)		59.6 ± 76.4	
Age				0.27		0.28
<60 y	126	98 (35)	28 (10)		73.2 ± 50.4	
≥60 y	154	110 (39.3)	44 (15.7)		79.4 ± 45.6	
Lesion				0.00^a^		0.00^a^
Vocal polyp	55	55 (19.6)	0 (0)		23.3 ± 11.6	
LPLs	102	93 (33.2)	9 (3.2)		61.1 ± 25.9	
LSCC	123	60 (21.4)	63 (22.5)		113.0 ± 42.5	
Differentiation				0.08		0.06
Well	28	18 (14.6)	10 (8.1)		99.9 ± 37.3	
Moderate+poorly	95	42 (34.1)	53 (43.1)		116.8 ± 43.3	
Smoking index				0.33		0.71
<400	166	127 (45.4)	39 (13.9)		74.9 ± 42.9	
≥400	114	81 (28.9)	33 (11.8)		73.1 ± 39.1	
Drink habit				0.15		0.09
None	179	138 (49.3)	41 (14.6)		75.7 ± 50.8	
Drink	101	70 (25)	31 (11.1)		77.9 ± 42.3	
TNM stage				0.001^a^		0.24
I to II	25	20 (16.3)	5 (4.1)		104.1 ± 26.5	
III to IV	98	40 (32.5)	58 (47.2)		115.2 ± 45.5	
Lymph nodes				0.001^a^		0.006^a^
Negative	66	44 (35.8)	22 (17.8)		103.3 ± 28.1	
Positive	57	16 (13)	41 (33.4)		124.2 ± 52.7	
LVD in LSCC				0.001^a^		0.002^a^
Low	54	35 (28.5)	19 (15.5)		91.4 ± 19.4	
High	69	22 (17.8)	47 (38.2)		111.4 ± 30.1	

HMGB1: high mobility group box protein 1; LPLs: laryngeal precursor lesions; LSCC: laryngeal squamous cell carcinoma; LVD: lymphatic vessel density. The data of the HMGB1 expression and CD163^+^ M2 macrophage density were analyzed using the *χ*^2^ test and Student's *t*-test, respectively (except for lesion variables); the data of lesion variables were analyzed by ANOVA with Dunnett's post hoc test. ^a^*P* < 0.05, statistical significance.

## Data Availability

All data generated or analyzed during this study are included in this published article.

## Abbreviations

HMGB1: High mobility group box protein 1
VP: Vocal polyp
LPLs: Laryngeal precursor lesions
LSCC: Laryngeal squamous cell carcinoma
LVD: Lymphatic vessel density
ECBM: Endothelial cell basal medium MV2.
